# Occurrence of Aflatoxin M_1_ in Dairy Products in Southern Italy

**DOI:** 10.3390/ijms9122614

**Published:** 2008-12-12

**Authors:** Maria Teresa Montagna, Christian Napoli, Osvalda De Giglio, Roberta Iatta, Giovanna Barbuti

**Affiliations:** Department of Biomedical Science and Human Oncology, University of Bari, Piazza G. Cesare, 11 - 70124 Bari, Italy. E-Mails: c.napoli@igiene.uniba.it (C. N.); osvaldadegiglio@tiscali.it (O. G.); iroberta@hotmail.com (R. I.); gbarbuti@dimo.uniba.it (G. B.)

**Keywords:** Aflatoxin M_1_, dairy products, food safety

## Abstract

A screening survey of the presence of aflatoxin M_1_ (AFM_1_) was carried out on 265 samples of cheese made from cow, buffalo, goat, sheep, sheep-goat milk collected in the Apulia region (Southern Italy). Selected samples included unripened, medium and long-term ripened cheeses. AFM_1_ was found in 16.6% of the analyzed samples. The highest positive incidence was for medium and long-term ripened cheeses, especially those made from sheep-goat milk, while buffalo cheeses tested consistently negative. Our results show that the level of contamination by AFM_1_ in dairy products from Apulia Region are lower than in other Italian and European regions. Moreover, it is important to underline that a common European norm concerning the AFM_1_ threshold limits for dairy products is still lacking.

## 1. Introduction

Aflatoxins are toxic metabolites, generally produced by *Aspergillus flavus, A. parasiticus* and *A. nomius* [1, 2]. They can have immunosuppressive, mutagenic, teratogenic and carcinogenic effects, especially on the liver [2, 3]. The best known are B_1_, B_2_, G_1_, G_2_, that are ingested by animals in contaminated pellets and forage; aflatoxin B1 (AFB_1_) is notoriously the most toxic of these metabolites. When ingested by dairy animals, the metabolite is biotransformed at the hepatic level by microsomial cytochrome P450 into aflatoxin M1 (AFM_1_) [4]. It is then excreted in this form in the milk used for human consumption and, thanks to its affinity for casein, is also present in dairy products [2, 5, 6]. It has been demonstrated that the concentration of AFM_1_ in cheese can also depend on the technology used in the production process, on the type of cheese and on the water content in the final product [2, 6, 7, 8]. Finally, although cheese is not a good growth substrate due to the low content of carbohydrates, the risk of synthesis of aflatoxins B_1_, B_2_, G_1_ and G_2_ by contaminating fungi or due to the use of contaminated powdered milk should not be underestimated [9].

The risk posed by aflatoxins has been faced in different ways in different countries. In Europe, the maximum tolerated levels of AFM_1_ in milk and dairy products were regulated firstly by Reg. CE 2174/2003 [10] that modified Reg.CE 466/2001 [11], and then by Reg. 1881/2006 [12]. In accordance with these norms, the product to be screened is milk, in which the AFM_1_ concentration must not exceed 0.05 μg/kg (= 0.05ppb = 50 ppt), while dairy products must be obtained using milk conforming to the above AFM_1_ limits. It has not been possible to set threshold limits for dairy products due to the difficulty of identifying a standardized milk-dairy product conversion factor. In fact, there are few data available in the national and international literature regarding the concentration factors in the different dairy products, and these data are dated and poorly applicable to national cheese-making processes [13, 14, 15, 16].

In Italy, after the outbreak of maize contamination by AFB_1_ that occurred in 2003, and the subsequent finding of AFM_1_ in milk [17], the Ministry of Health established a maximum permissible value of 0.45 μg/kg (= 0.45 ppb=450ppt) of mycotoxin in hard, long term ripened cheeses [18]. This value, although considered at the time as an interim measure to deal with the crisis, has not since been updated and some authors have used it as the threshold value for AFM1 contamination in cheeses [19]. However, it is much higher than those adopted in other states: 0.25 μg/kg in Switzerland, Iran and Turkey, 0.20 μg/kg in Holland [1, 20, 21]. A common European norm applicable in all the member states is still lacking.

As regards the analytical methods to be used to search for AFM_1_ in cheeses, Italian law establishes that the Enzyme-Linked Immunosorbant Assay (ELISA) [22] can be used for screening and High-Performance Liquid Chromatography (HPLC) for confirmation purposes [18]. Many authors have used the ELISA method and consider it a valid test to search for aflatoxins [2, 20, 23–28].

The aim of the present work was to carry out screening for the presence of AFM_1_ in cheeses produced in the Apulia Region, to contribute to making a proper assessment of the health risk present in our nation and planning any necessary prevention measures.

## 2. Methodology

Tests for AFM1 were made on 265 samples of cheeses collected at different sales centres distributed evenly over the Apulian territory: 89 samples were unripened (<1 month), 88 medium term (1–3 months) and 88 long term ripened (3–12 months) cheeses. This classification was adopted in accordance with the definitions of the Italian Ministry for Agricultural and Forest Policies [29]. In view of the frequency of consumption of the different products in our region, the selected samples included sheep (n = 94), cow (n = 92), buffalo (n = 51), mixed sheep-goat (n = 16) and goat (n = 12) cheeses.

The research protocol consisted of screening for AFM_1_ using the ELISA test and a confirmation HPLC test in cases of contamination levels exceeding 0.45 μg/kg in hard, long term ripened cheeses [18] and 0.25 μg/kg in all the other cheeses, as suggested by other authors [20, 26, 30]. Since no levels of AFM1 exceeding these thresholds were identified in our study, we did not need to perform the HPLC method and so we describe only the ELISA method adopted.

The *I’screen* AFLA M_1_ kit (Tecna, Trieste, Italy) – detection limit of 0.037 ppb (μg/kg) in cheese, certified by manufacturer - conforming to the En ISO 14675:2003 norms, was used. Two grams of cheese were homogenized for 15 minutes with a Stomaker (International PBI, Milan, Italy), with addition of dichloromethane (15 mL). After filtration through Whatman paper filters, part of the solvent (3.75 mL) was allowed to evaporate at 60°C under nitrogen flow; the residue was suspended on the extraction buffer (750 μL), vortexed for 30 seconds, resuspended in hexane (750 μL), and again vortexed for 1 minute. After centrifugation for 15 minutes at 2,000 g and removal of the hexane, 50 μL of solution were extracted and added to 200 μL of dilution buffer. This compound was subjected to quantitative immunoenzymatic assay.

A hundred μL of standard solution and the previously prepared samples were added to micro titer wells and then incubated at room temperature for 45 minutes. At the end of incubation, the liquid in the wells was poured out, and the micro well holder was tapped upside down on an absorbent paper to remove the remainder of the liquid. The wells were washed four times with washing buffer, and then the liquid in them was poured out. A hundred μL of the diluted enzyme conjugate was added to each well, and they were gently shaken. The wells were incubated for 15 minutes. The wells were washed four times with washing buffer, and then the liquid in them was poured out. A hundred μL of developing solution were added to each well and then incubated for 15 minutes. Following the addition of 100 μL of the stop reagent to each well, absorbance was measured spectrophotometrically at 450 nm (ETI System Fast Reader ELX, Biotek, US). The data obtained from the standards and samples were evaluated using the spreadsheet downloadable at www.tecnalab.com to calculate sample concentration.

A part from the kit control, to check the quality for the level of AFM_1_ contamination by *I’screen* AFLA M_1_ kit, two samples at known concentration (20 and 50 ppt, evaluated by six different laboratories by HPLC) were tested. *I’screen* AFLA M_1_ kit confirmed the known concentrations.

The χ^2^ test was used to assess the significance of comparisons of the positive percentages of cheeses at different stages of ripening and milk origin, setting the significance level at p<0.05. The statistical software package SPSS version 16 Italian was employed.

## 2. Results

AFM_1_ was found in 16.6% of the cheeses tested (Table 1): 31.3% of these were sheep-goat cheeses, 27.2% cow, 16.7% goat and 12.8% sheep cheeses (Table 1), with no significant differences (p>0.05). All the samples of buffalo milk cheese were consistently negative.

Medium term ripened cheeses were the most frequently contaminated (19.3%), followed by long term (17.1%) and lastly unripened (13.5%) cheeses. No significant differences were found between the contamination levels at the different stages of ripening (p>0.05).

Overall, the AFM_1_ values ranged from 50 to 250 ppt (Table 2). Specifically, the concentrations of AFM_1_ in goat cheese ranged from 90 to 250 ppt, in cow cheese from 50 to 160 ppt, in sheep cheese from 50 to 215, in sheep-goat cheese from 55 to 140 ppt.

## 3. Discussion and Conclusions

In this study measurable levels of AFM_1_ were present in 16.6% of the dairy products examined, all below the threshold limits considered for the present study [18, 20, 26, 30]. With regards to the stage of ripening of the positive samples, the most affected samples were those of medium term (19.3%) and long term ripened cheeses (17.1%), although comparison with the data on unripened cheeses (13.5%) did not yield any statistically significant difference. Our data are in line with those reported in the literature, whereby AFM_1_ concentrations increase with the stage of ripening and so medium and long term cheeses are at greater risk [19, 30]. In fact, AFM_1_ has a high affinity for milk proteins and therefore during the cheese-making process ripening does not reduce the levels of AFM_1_ but on the contrary increases them, due to water loss [9, 31, 32].

Notably, buffalo cheese samples were consistently negative, while mixed sheep-goat and cow cheeses were more contaminated (31.3% and 27.2%, respectively) than pure sheep or goat cheeses (12.8% and 16.7%, respectively). It is difficult to explain the greater contamination of mixed sheep-goat cheeses as compared to pure sheep or goat cheeses, especially because sheep and goat are generally fed fresh fodder. This difference might be linked to the single samples analyzed and the processing techniques adopted rather than to the origin of the milk (for example, the combined heat and acid treatment used for the production of ricotta cheese was shown to alter the structure of whey proteins to the point where they lost their ability to bind the toxin [9]).

The data on cow cheese, on the otherhand, are not out of ordinary. In fact, cows are generally fed stored fodder and concentrates (cereals, protein meal, integrated minerals), that are notoriously more prone to contamination by AFB_1_ [33]. This suggests that the levels of milk contamination do not only reflect peculiarities of the dairy species but also of the feed administered and the length of fodder storage, where the environmental conditions (humidity, temperature etc) foster fungal growth. In this context, some authors have reported that milk produced during the winter presents much higher levels of AFM_1_ than those present in the summer. It can therefore be assumed that a lesser consumption of these feeds and the chance to allow the animals to graze freely could contribute to reduce the level of contamination in milk [5, 26, 34]. In fact, in Apulia there is ample grazing land due to the type of territory and the favourable climatic conditions, so there is less consumption of stored fodder and thus the levels of contamination by AFM_1_ are lower than in other Italian and European regions [2, 5, 15, 19, 28, 34–36].

Moreover, in the present study, no AFM_1_ levels were >0.25 μg/kg; on the contrary, previous studies showed levels of AFM1 >0.25 μg/kg: Baskaya *et al*. [2] reported that 22.4% of all analyzed cheese samples were above this limit; Aycicek *et al*. [36] found that 19% of cheese samples had AFM_1_ levels greater than 0.25 μg/kg; in another study performed by Kamkar *et al*. [37] it was found that almost 60.6% of the contaminated samples exceeded the above mentioned limit.

As to the lack of contamination of buffalo cheeses, a hypothesis – not confirmed in the present study - could be given by data in the literature. Some authors have shown that strains of lactobacilli (present in higher concentrations in buffalo than in cow milk) are able to bind AFB_1_ and AFM_1_, thus decontaminating the milk secreted [38, 39, 40].

In conclusion, considering that for various reasons many regions are obliged to feed dairy animals on stored forage or industrially produced pellets, it is important to reduce the occurrence of toxins (AFB_1_) in feedstuff and take prophylactic measures to prevent factors enhancing toxin production. [41]. These factors include environmental temperature, humidity, and moisture content of the feed as well as pH and mechanical damage to the grain affecting mould production [41].

Moreover, since AFM_1_ is well known to be mutagenic and carcinogenic, international regulations ensuring a minimal presence of this aflatoxin in cheeses are needed. In fact, by having a common European norm concerning the AFM_1_ threshold limits for dairy products will it be possible to better guarantee the distribution of safer, healthier foods, particularly in light of the current norms on internal controls and HACCP [42].

## Figures and Tables

**Table 1.** Presence of AFM_1_ in 265 cheese samples.

TYPE OF CHEESE	N positive samples / Total						Positive samples/Total	
	Unripened		Medium term ripened		Long term ripened			
	n/N	%	n/N	%	n/N	%	n/N	%
**sheep**	5/31	16.1	3/32	9.4	4/31	12.9	12/94**	12.8
**cow**	5/31	16.1	10/30	33.3	10/31	32.3	25/92**	27.2
**buffalo**	0/17	0.0	0/17	0.0	0/17	0.0	0/51**	0.0
**sheep-goat**	1/6	16.7	3/5	60.0	1/5	20.0	5/16**	31.3
**goat**	1/4	25.0	1/4	25.0	0/4	0.0	2/12**	16.7
**TOTAL**	12/89*	**13.5**	17/88*	**19.3**	15/88*	**17.1**	**44/265**	**16.6**

* No significant difference between the contamination level at different terms of ripening

** No significant difference between the contamination level at different type of cheese

**Table 2.** Mean values and range of AFM_1_ in positive cheese samples of different type and at different terms of ripening.

**Type of cheese**	**Ripening term**	**Mean AFM1values** (ppt)	**Range of AFM1** (ppt)
cow	long	73.0	50–160
	medium	70.7	50–114
	short	81.4	50–158
buffalo	long	0.0	0–0
	medium	0.0	0–0
	short	0.0	0–0
goat	long	0.0	0–0
	medium	250.0	250
	short	90.0	90
sheep	long	111.3	50–215
	medium	121.7	55–190
	short	105.0	60–170
sheep-goat	long	75.0	75
	medium	85.0	55–140
	short	50.0	50
	TOTAL	88.6	50–250
